# A pilot study on the immune cell proteome of long COVID patients shows changes to physiological pathways similar to those in myalgic encephalomyelitis/chronic fatigue syndrome

**DOI:** 10.1038/s41598-023-49402-9

**Published:** 2023-12-12

**Authors:** Katie Peppercorn, Christina D. Edgar, Torsten Kleffmann, Warren P. Tate

**Affiliations:** Division of Health Sciences, Department of Biochemistry, School of Biomedical Sciences, University of Otago, Dunedin, 9016 New Zealand

**Keywords:** Biochemistry, Immunology, Molecular biology, Physiology, Medical research

## Abstract

Of those infected with severe acute respiratory syndrome coronavirus 2 (SARS-CoV-2), ~ 10% develop the chronic post-viral debilitating condition, long COVID (LC). Although LC is a heterogeneous condition, about half of cases have typical post-viral fatigue with onset and symptoms that are very similar to myalgic encephalomyelitis/chronic fatigue syndrome (ME/CFS). A key question is whether these conditions are closely related. ME/CFS is a post-stressor fatigue condition that arises from multiple triggers. To investigate the pathophysiology of LC, a pilot study of patients (n = 6) and healthy controls (n = 5) has used quantitative proteomics to discover changes in peripheral blood mononuclear cell (PBMC) proteins. A principal component analysis separated all long COVID patients from healthy controls. Analysis of 3131 proteins identified 162 proteins differentially regulated, of which 37 were related to immune functions, and 21 to mitochondrial functions. Markov cluster analysis identified clusters involved in immune system processes, and two aspects of gene expression-spliceosome and transcription. These results were compared with an earlier dataset of 346 differentially regulated proteins in PBMC’s from ME/CFS patients (n = 9) analysed by the same methodology. There were overlapping protein clusters and enriched molecular pathways particularly in immune functions, suggesting the two conditions have similar immune pathophysiology as a prominent feature, and mitochondrial functions involved in energy production were affected in both conditions.

## Introduction

Infection with severe acute respiratory syndrome coronavirus 2 (SARS-CoV-2) results in a post-viral condition, long COVID (LC), in a significant proportion (~ 10%) of those affected^1^. With now over 772 million cases of SARS-CoV-2 infection worldwide as at November 22nd 2023^2^ there will be an estimated ~ 80 million cases of LC. Long COVID is a chronic debilitating disease arising from this unique viral trigger. The diagnosis of LC is made three months from initial infection when the condition persists for more than eight weeks^3^. Although LC is recognized as heterogeneous, with some patients suffering from ongoing organ damage, at least 50% have a post-viral fatigue condition^4,5^ with onset and symptoms very similar to myalgic encephalomyelitis/chronic fatigue syndrome (ME/CFS), the collective term for the post-viral or post-stressor fatigue condition arising from such multiple triggers^6^. A recent paper has defined four clinical phenotypes amongst LC patients with the ME/CFS-like fatigue condition the dominant phenotype^5^. Children, adolescents, and adults can all be affected by ME/CFS and LC with impacts including the inability to pursue education, work or normal life activities^7,8^. In contrast to LC, ME/CFS is diagnosed formally with a clinical case definition if symptoms persist for at least six months^9^ while LC is diagnosed after three months^3^, but for most ME/CFS patients diagnosis has been made much later, when their condition has become well established and other illnesses have been eliminated^10^. For those patients with ongoing ME/CFS, the debilitating condition is lifelong^11^ with a resulting heavy burden on families and with severe economic and social impacts. For both conditions, symptoms reported are numerous (estimated to be > 100) and the vast majority of those of LC overlay with those of ME/CFS, with only a small number of differences, perhaps relating to the unique effects from the triggering SARS-CoV-2 virus. Nevertheless, the key commonest symptoms identified by the WHO when deriving a clinical case definition for LC: fatigue, brain fog (cognitive defects), activity related post exertional malaise, unrefreshing sleep, pain, and other neurological symptoms are shared with ME/CFS in the most widely accepted clinical case definitions for this condition.

Onset of several diseases have also been attributed to infection with SARS-CoV-2, such as type 2 diabetes, and dysautonomia, especially postural orthostatic tachycardia syndrome (POTS)^4,12^. Increasingly, with focus on LC within the first few months of onset some commentators have proposed that if the post-viral fatigue condition of LC patients lasts beyond one year it ‘becomes’ ME/CFS. The major clinical phenotype of LC with fatigue symptoms may represent either a specific example of ME/CFS facilitated from the single triggering SARS-COV-2 virus in susceptible people and exhibiting the same dysfunctional physiology generally as in ME/CFS patients, or it may exhibit significantly different molecular changes arising from the specific characteristics of the SARS-C0V-2 infection. This study aims to explore this question. If there were fewer cases of COVID-19 worldwide, as with the first SARS CoV-1 outbreak in 2003, LC would most likely have been classified as ME/CFS like the other 75 boutique outbreaks of probable viral infections reported since about 1930^13^.

We have proposed a model to explain the complex dysfunctional physiology for both ME/CFS and LC^14^. A key feature is that in susceptible people the normal transitory immune/inflammatory response of the peripheral system to infection or stress does not resolve quickly as in most people but becomes chronic and that leads to a cascade effect, with involvement of the brain and its immune system, and other components of the central nervous system. The disturbed functions of the CNS result in multiple neurological symptoms, and in poor brain regulation of body physiology. A number of studies have documented chronic dysregulation of the immune cell function involving multiple cell types, and cytokines in ME/CFS^15–21^, and now more recently in LC^22–25^. In ME/CFS, multiple disturbances have been reported in the molecular homeostasis of the transcriptomes^26–28^, proteomes^29–31^, and DNA methylomes^32–36^ of peripheral blood mononuclear cells (PBMC) that include lymphocytes (T cells, B cells, NK cells) and monocytes^37^, but not as yet in LC.

This current study analysed the PBMC proteomes of post-viral fatigue LC patients whose illness had lasted for one year, compared with age/sex matched healthy controls. It aimed to identify specific areas of cellular physiology that were still dysfunctional a year on from the patients’ initial SARS-CoV-2 infection. Groups of proteins involved with the immune response, and gene expression (spliceosome and transcription) and mitochondrial function were shown to be differentially regulated in LC patients. We have previously published data on the differentially regulated proteins in a ME/CFS cohort with an average disease duration of 16 years, using the same mass spectrometry strategy^29^, and here we compared the two datasets to identify common or distinct features. We hypothesised that there would be similar molecular pathways affected that would contribute to and explain the closely overlapping symptoms and pathophysiology of both long COVID and ME/CFS. Despite the significant difference in the disease durations of the two groups of patients (1 year vs an average of 16 years) many common effects were identified. Therapeutic targeting of the immune response/inflammatory pathways may be beneficial for treatment of both diseases.

## Results

We first analysed the proteomes of six LC patients and five age/sex matched healthy controls (HC) by Sequential Window Acquisition of all Theoretical Fragment Ion Spectra-Mass Spectrometry (SWATH-MS) to identify proteins differentially regulated in LC patients. We then compared these data with a study of nine ME/CFS patients and age/sex matched controls that were also analysed by SWATH-MS. The ME/CFS patients were all recruited and analysed before the onset of the pandemic in New Zealand in 2020. The demographics and characteristics of both cohorts are shown in Table 4.

### The spectral library

A comprehensive spectral library containing the peptide spectra of all of the identified proteins in a pool of the samples from all LC and HC subjects was generated through data-dependent acquisition (DDA) mass spectrometry. The pooled sample contained an equal amount of sample from each patient and control subject. The DDA mass spectrometry of the pre-fractionated pooled sample identified 3566 protein groups at a confidence interval of ≥ 99 and an FDR of q ≤ 0.01, which were integrated into the spectral library. After aligning the Data Independent Acquisition (DIA) SWATH-MS data to the spectral library, 3131 proteins and 11,127 individual peptides were used for subsequent quantification between the patient and control groups based on an FDR for a peak matching of q ≤ 0.01 in at least one sample.

### Differentially regulated proteins in long COVID patients

**Figure 1:**
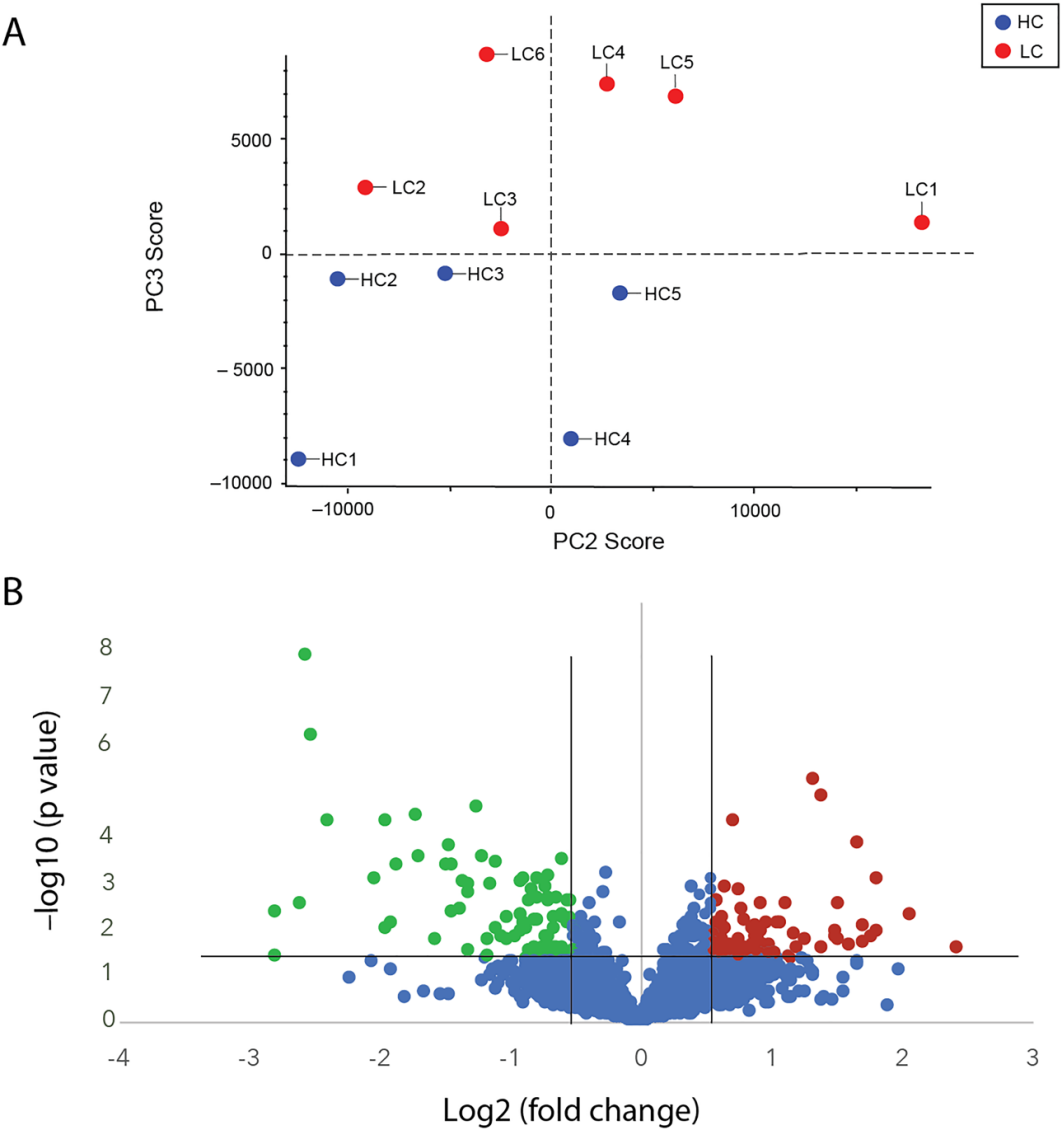
Abundance profile of long COVID patients differed from healthy controls. (**A**). A principal component analysis detected sufficient differences in the dataset to segregate the long COVID patient group from healthy controls in principle component (PC) 3 (6.8% of differences). (**B**). Volcano plot showing significantly down- (green) and up-regulated (red) proteins. Unchanged or non-significantly changed proteins are blue. The significance thresholds were a fold change of at least 1.5 (log2(fold change) ≤ -0.58 or ≥ 0.58) at a p value of 0.05 or lower (-log10(*p* value) ≤ 1.3) are indicated by the black lines. Abbreviations; LC—long COVID, HC—healthy control.

A principal component analysis (PCA) on all quantitative protein data and all samples detected sufficient differences to segregate all LC patients from the HC group in principal component 3 (Fig. 1A). Using the average of all the peptide intensities of each protein for quantification, 162 of the 3131 proteins in the spectral library were identified as being differentially regulated in the LC samples (minimum 1.5-fold change (log2(FC) ≤  − 0.58 or ≥ 0.58) and a significance threshold of *p* ≤ 0.05 (− log10(*p*-value) ≥ 1.3). The fold change decision for inclusion may vary according to the biological system (cell or human patients) and whether such a fold change is likely to affect the biology—particularly relevant for human studies. A fold change minimum of 1.5 was selected here based on our previously published proteomic studies on samples from human patients and human neural and mammalian neural cells^29,38,39^. This inclusion limit returned enough proteins to perform a network analysis on proteins of interest biologically.

There were 79 proteins down-regulated (green) and 83 up-regulated (red), as illustrated in the volcano plot (Fig. 1B). A list of all the proteins that were significantly differentially regulated is found in Supplementary Table S1.

**Figure 2:**
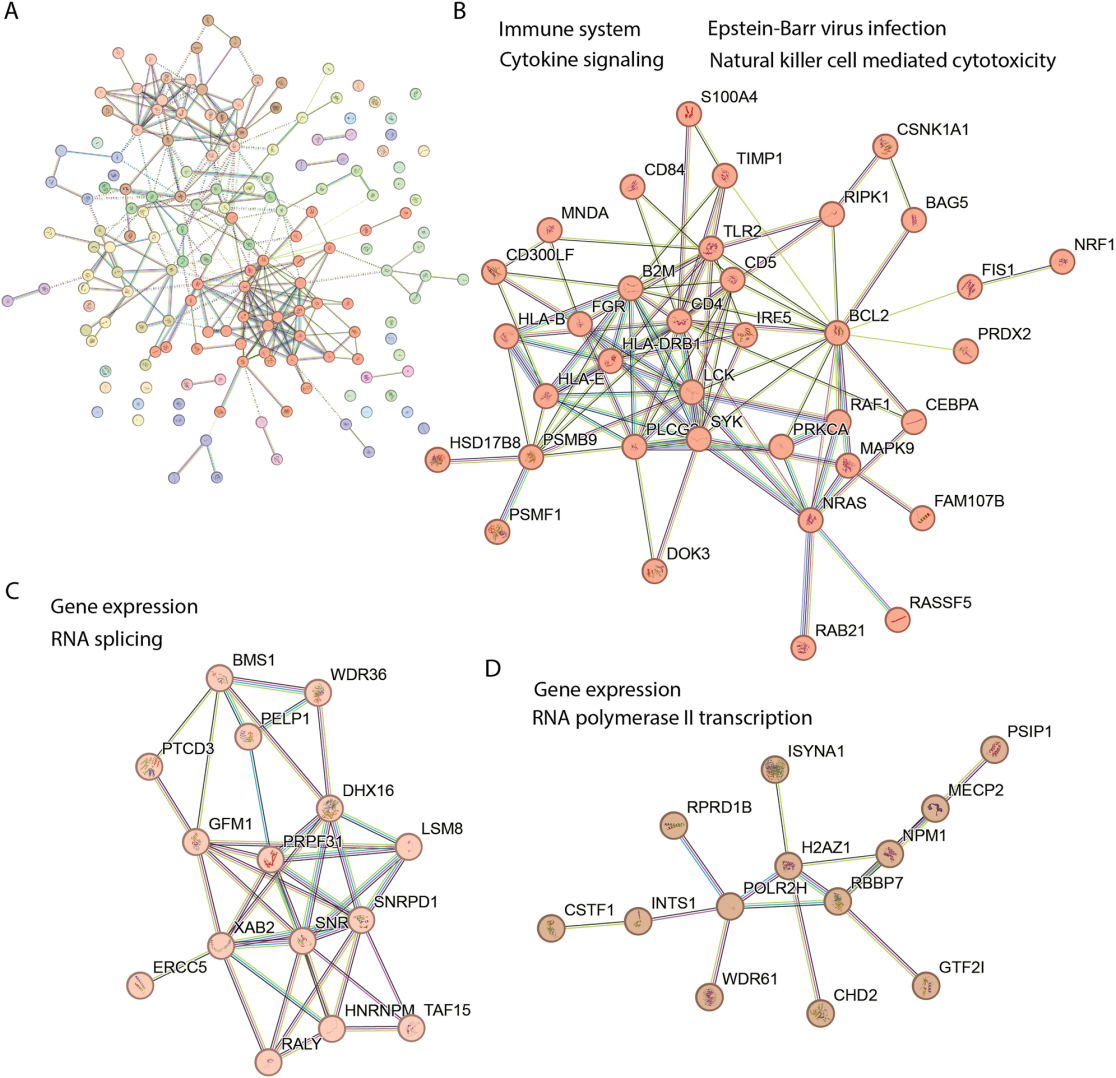
Differentially regulated proteins in long COVID patients clustered into three groups. (**A**). Graphical representation of a STRING (v12) network of 162 differentially regulated proteins after Markov Cluster Algorithm (MCL) analysis with an inflation parameter of 1.5. The colours in the full network (**A**) indicate the clusters of functionally associated proteins. The three main clusters shown in (**B**–**D**) are enriched in proteins associated with the following functional GO annotations: B. immune system (Reactome HSA-168256), Epstein–Barr virus infection (KEGG hsa05169), Natural killer cell mediated cytotoxicity (KEGG hsa04650) and Cytokine Signaling in Immune system (Reactome HSA-1280215); (**C**). gene expression (GO molecular process GO:0,010,467) and RNA splicing (GO:0,008,380); (**D**). gene expression and RNA Polymerase II Transcription (GO molecular process HSA-73857). The protein symbol is used to identify each protein and the full names can be found in supplementary material Table S1.

A STRING (v12) functional network analysis^40^ on the 162 differentially regulated proteins showed numerous protein nodes with direct and indirect connections (edges) to other proteins within the set of differentially regulated proteins (Fig. 2A) and highlighted enrichment of proteins associated with a number of Gene Ontology (GO) terms (Table 1, and supplementary Table S2).

**Table 1:** Reactome and KEGG pathways most significantly represented by protein nodes with a high level of connectivity (10 or more first level edges to other nodes).

Reactome ID	Term description	OGC	BGC	Strength	FDR
HSA-168256	Immune System	37	1979	0.36	4.50E-04
HSA-168249	Innate Immune System	27	1041	0.50	6.81E-05
HSA-1280215	Cytokine Signaling in Immune system	19	706	0.51	1.60E-03
HSA-2172127	DAP12 interactions	7	39	1.34	6.37E-05
HSA-5663205	Infectious disease	31	917	0.61	6.86E-08
HSA-9679506	SARS-CoV Infections	17	411	0.70	6.37E-05
KEGG ID					
hsa04650	Natural killer cell mediated cytotoxicity	8	120	0.91	1.70E-03
hsa04664	Fc epsilon RI signaling pathway	6	65	1.05	2.80E-03
hsa05169	Epstein-Barr virus infection	9	192	0.76	3.50E-03
hsa05170	Human immunodeficiency virus 1 infection	12	203	0.86	6.93E-05

*OGC* observed gene count,* BGC* total background gene count in that category, FDR-false discovery rate. Strength measures the confidence score of interactions between proteins or genes calculated by the STRING database tool. It quantifies the reliability of associations, reflecting the likelihood of true functional connections > 0.4 is considered significant.

The full network of the 162 differentially regulated proteins (Fig. 2A) comprised 16 protein nodes with first level connections to ten or more differentially regulated proteins. These 16 protein nodes listed here are indicated by their protein symbol and their number of first level interactions is shown in brackets; B2M (16), BCL2 (20), CD4 (22), GFM1 (14), HLA-DRB1 (10), HNRNPM (10), LCK (12), NMP1 (16), NRAS (12), PLCG2 (10), PSMB9 (12), SNRPB (10), SNRPD1 (12), SYK (14), TLR2 (10) and UBQLN2 (11) (see Supplementary Table S1 for protein names). This demonstrates a high level of functional association and intra connectivity (betweenness centrality) among the regulated proteins. An enrichment analysis shown in Table 1 highlights a selection of the Reactome and KEGG pathways that suggest the correlations of subsets of these proteins in immune system functions^41^. DAP12 is a DNAX-activating protein of 12 kDa that acts as a signaling adapter protein expressed in Natural Killer (NK) cells and myleoid cells participating in innate immune responses^42^. Terms related to responses to SARS-COV-2, Epstein-Barr virus and HIV infections were prominent (see supplementary Table S2 for the full list of enriched GO terms).

Table 1 indicates that changes in immune system-related proteins feature significantly in the data from LC patients. The STRING network analysis tool returns the number of proteins/genes from the set of significantly differentially regulated proteins, that are associated with any given GO term. This is the ‘observed gene count (OGC)’, and the ‘background gene count (BGC)’ is the total number of proteins/genes for that category. The broad generalised Reactome (HSA-168256) category 'Immune System' was significantly enriched with 37 or 23% of the differentially regulated proteins (strength 0.36, FDR 0.00045). The immune system’s 37 differentially regulated proteins are listed here: AP2S1, ATP6V1G1, B2M, BCL2, CD300LF, CD4, DOK3, DYNC1I2, FGR, GMFG, HLA-B, HLA-DRB1, HLA-E, IRF5, KPNA3, LCK, MAPK9, MNDA, NDUFC2, NRAS, NUP93, PAFAH1B2, PLCG2, POLR2H, PSMB9, PSMF1, PTGES2, RAB6A, RAF1, RIPK1, SERPINB10, SYK, TIMP1, TLR2, TRIM22, TRIP12 and UBA3. The more specific term 'cytokine signaling in immune system' (Reactome HSA-1280215) included 19 proteins (strength 0.51, FDR 0.001) listed here: CD4, BCL2, B2M, HLA-DRB1, LCK, NRAS, PSMB9, PLCG2 and SYK (all first level interactors for ≥ 10 other differentially regulated proteins), while TIMP1 (6), KPNA3 (4), NUP93 (4), PSMF1 (1), UBA3 (3), HLA-E (9), TRIM22 (2), MAPK9 (5), HLA-B (5) and IRF5 (4) also interact with multiple proteins (numbers of interacting proteins shown in brackets), giving weight to the association of immune system dysregulation in LC.

Then Markov Cluster Algorithm (MCL) analysis^43^ with an inflation parameter of 1.5 was performed to highlight modules of associated protein nodes within the complex network that show stronger edge interactions to each other than to the rest of the network and therefore allow a more sensitive pathway enrichment analysis by reducing network complexity. Three major clusters were identified that contained 10 or more protein nodes among the 162 differentially regulated proteins in the LC patients (Fig. 2A). One cluster (Fig. 2B) is broadly associated with the immune response, and the other two with gene expression (spliceosome—Fig. 2C), and transcription (Fig. 2D). See supplementary Table S3 for complete cluster analysis with the protein names and their predicted functions.

Interestingly, 17 proteins (out of a possible 411 in the category) in Table 1 are enriched from the Reactome (HSA-9679506) category 'SARS-CoV Infections' (Strength 0.7, FDR 6.37E-05); RIPK1, TLR2, MAN2A1, CSNK1A1, NPM1, SNRPD1, NUP93, SYK, HLA-E, RBBP7, HLA-B, SNRPB, CHMP2A, AP2S1, PTGES3, PLCG2 and B2M. The changed regulation of some of the proteins discussed above that are associated with ‘Immune System’ in general may have originated first in response to infection, in this case infection with the 'SARS-CoV-2 virus’, but persisted with the onset of LC.

To identify potential specific pathways involved in LC, a search was made for any specific GO terms where at least 25% of proteins from the small background gene counts (BGC) were differentially regulated in the LC dataset. More highly specialised GO terms describe specific molecular functions that involve fewer proteins i.e. these terms have lower BGC. Examples of GO terms where there are ≥ 25% proteins differentially regulated (OGC/BGC) include ‘antigen processing and presentation exogenous peptide antigen via MHC class 1b (2/4), ‘protection from NKC mediated cytotoxicity’ (2/6) and CD4 receptor binding (2/8). The GO terms satisfying this criterion and representing specific immune cell functions have been included along with the relevant protein symbols in supplementary Table S4.

### Comparison with an earlier study of ME/CFS patients

We reported a similar proteome study with a well characterized group of pre-pandemic ME/CFS patients in 2020 compared with age/sex matched healthy controls^29^. By contrast this patient cohort had been affected by ME/CFS on average for 16 years compared with each of the LC cohort for only 1 year. Although the female to male ratios were different in the ME/CFS study compared with the LC cohort used in this study, we found no PCA separation on the basis of gender or age in the ME/CFS cohort^29^, and the one male patient in the LC cohort here was within the centre of the LC patient cluster shown in Fig. 1A.

In the ME/CFS study, there were 346 differentially regulated proteins compared with the 162 proteins identified in the current LC study that met the same criteria (minimum 1.5-fold change and *p*-value ≤ 0.05) used for the selection in the LC study (Supplementary Table S5). Of these 153 were up-regulated and 193 down-regulated.

**Figure 3:**
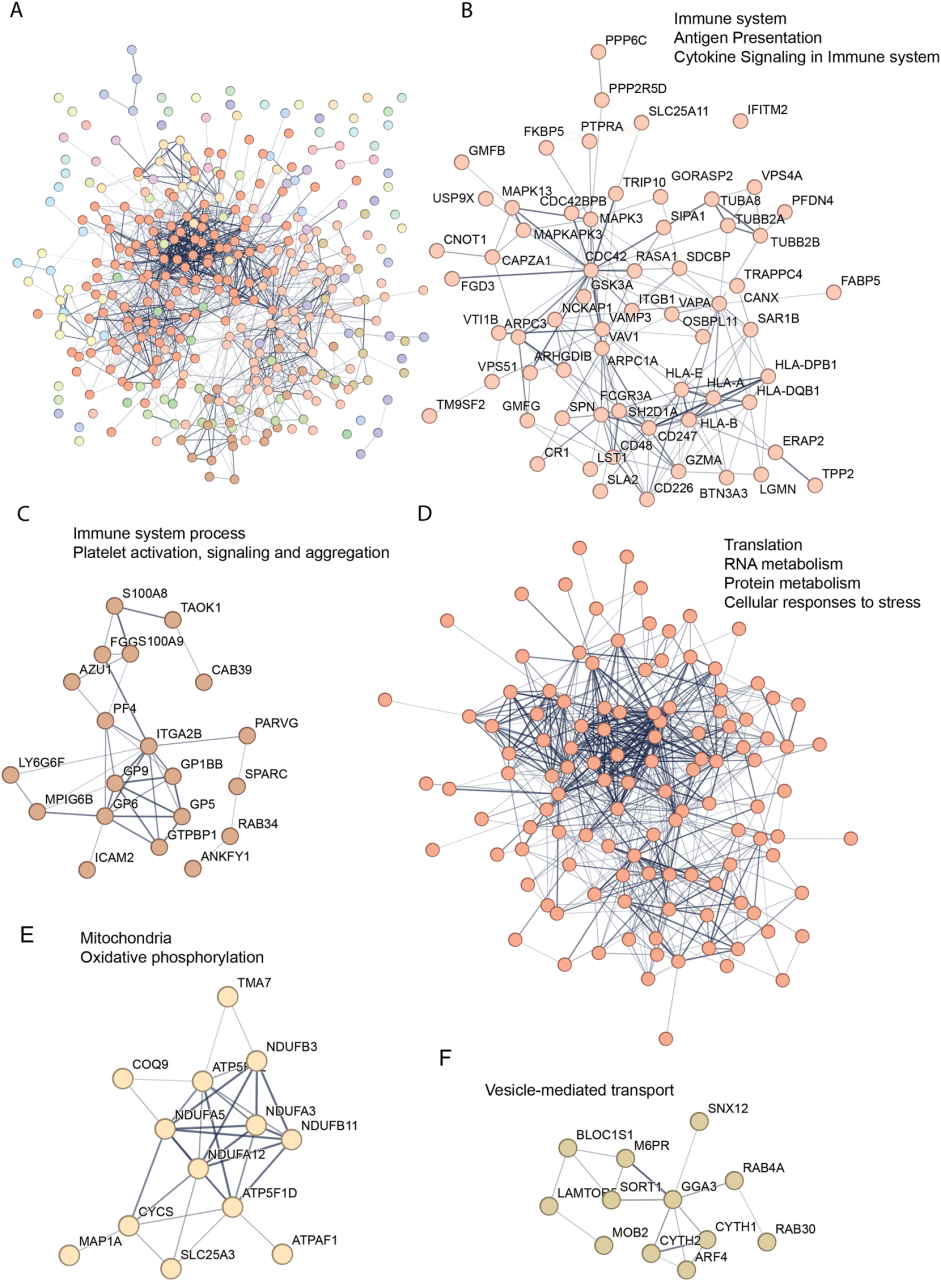
ME/CFS MCL cluster analysis of differentially regulated proteins. (**A**). Graphical representation of a STRING (v12) network of 346 differentially regulated proteins after MCL cluster analysis using an inflation parameter of 1.5. A. Entire protein network, each colour represents a cluster of functionally associated protein nodes. (**B**). 63 proteins involved with the immune system (Reactome HSA-168256), Antigen Presentation (Reactome HSA-983170) and Cytokine Signaling in Immune system (Reactome HSA-1280215). (**C**). 20 proteins associated with immune system process associated with Immune system process (Molecular process GO:0,002,376) and Platelet activation, signaling and aggregation (Reactome HSA-76002E). (**D**). 119 proteins associated with Translation (Reactome HSA-72766), RNA metabolism (Reactome HSA-8953854), Protein metabolism (Reactome HSA-392499) and Cellular responses to stress (Reactome HSA-2262752). (**E**). 13 proteins associated with the mitochondria (GO cellular component GO:0,005,739) and Oxidative phosphorylation (GO cellular process GO:0,006,119). F. 12 proteins associated with vesicle-mediated transport (GO biological process GO:0,016,192). Networks in A and D are too large to include protein symbols. The full protein names can be found in supplementary Table S5.

Using the most recent version of the STRING tool (v12) for consistency, an updated STRING functional network MCL cluster analysis of the regulated proteins from the previous ME/CFS study was carried out and revealed five clusters with 12 or more proteins (Fig. 3); two clusters were broadly associated with the immune system—antigen presentation and cytokine signalling (63 proteins—Fig. 3B) and immune system process—platelet activation, signalling and aggregation (20 proteins Fig. 3C), one with gene expression and metabolism—translation, RNA metabolism, protein metabolism and cellular response to stress (119 proteins—Fig. 3D), and smaller clusters associated with the mitochondria—oxidative phosphorylation (13 proteins—Fig. 3E and vesicle-mediated transport (12 proteins—Fig. 3F).

A comparison of the STRING pathway enrichment analysis outputs for the LC data against the ME/CFS dataset (re-analysed here at FC ≥ 1.5 with *p* ≤ 0.05) allowed for the identification of both common and distinct enriched functional categories or pathways between the two datasets. Twenty-two Reactome pathways and 5 KEGG pathways were common to both LC and ME (Supplementary Table S6). Supplementary Figure S1 shows a Venn diagram of these Reactome pathways and KEGG pathways that were common to the two datasets.

**Figure 4:**
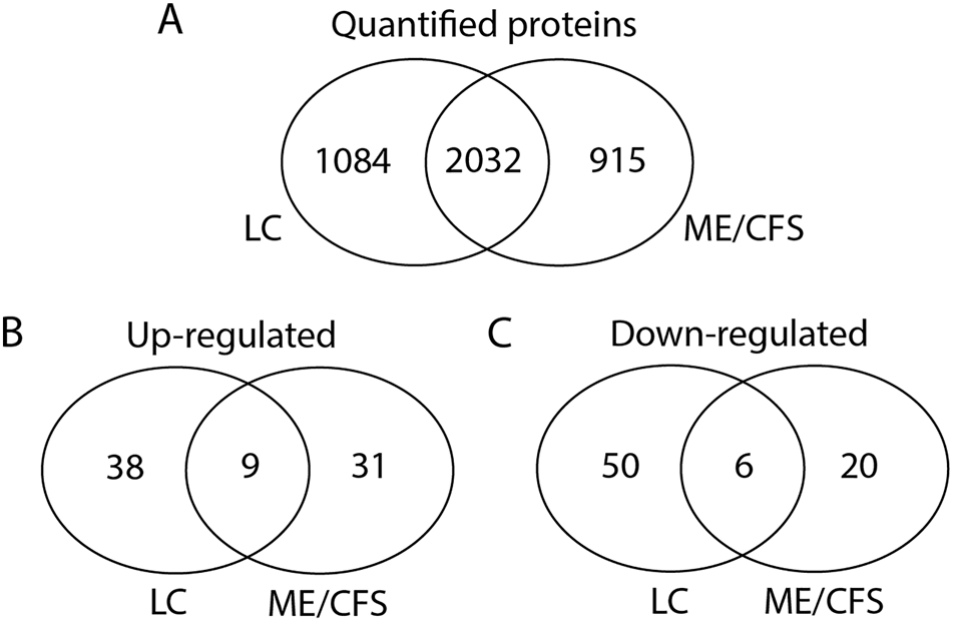
Comparison of the total number of quantified proteins and those differentially regulated in LC and ME/CFS. (**A**). Proteins identified and quantified in the LC and ME/CFS datasets that overlap or are present in only one dataset. (**B**). Differentially up-regulated proteins within the common 2032 proteins that are found in both LC and ME/CFS datasets or in only either LC or ME/CFS. (**C**). Differentially down-regulated proteins within the common 2032 proteins that are found in both LC and ME/CFS datasets or in only either LC or ME/CFS.

For a comparison of the proteins differentially regulated between the LC and ME/CFS datasets we first evaluated the overlap of proteins in the quantitated proteins dataset of both studies. As shown in the Venn diagram in Fig. 4A there were 2032 proteins identified in both datasets, with 1084 only in the LC study, and 915 only in the ME/CFS study. Of the differentially regulated proteins within the 2032 proteins common to both datasets, there were 47/83 of the LC up-regulated and 56/79 of the down-regulated proteins, and 40/153 of the ME/CFS up-regulated and 26/193 of the down-regulated proteins. Nine were up-regulated and six down-regulated common to both LC and ME/CFS data sets, as shown in the Venn diagrams in Fig. 4B and C.

The characteristics of those differentially regulated proteins that were detected in both data sets are shown in Table 2.

**Table 2:** Common proteins differentially regulated in the LC and ME/CFS groups.

Differentially regulated proteins in ME/CFS and long COVID patients		Fold change		Regulation
Protein symbol	Protein name	ME-CFS study	LC study	ME-CFS/LC
DNAJB11	dnaJ homolog subfamily B member 11 precursor	0.49	0.61	Down
HLA-B	major histocompatibility complex, class I, B precursor	0.61	0.17	Down
TPM1	tropomyosin alpha-1 chain	0.53	0.15	Down
GUCY1A1	guanylate cyclase soluble subunit alpha-3	0.5	0.60	Down
TST	thiosulfate sulfurtransferase	0.65	0.64	Down
PAFAH1B2	platelet-activating factor acetylhydrolase IB subunit beta	0.2	0.49	Down
PSMB9	proteasome subunit beta type-9 proprotein	1.68	1.82	Up
TMA7	translation machinery-associated protein 7	1.69	1.70	Up
RPL28	60S ribosomal protein L28	1.85	1.83	Up
PRDX2	peroxiredoxin-2	1.36	1.70	Up
ARHGDIB	rho GDP-dissociation inhibitor 2	1.54	1.83	Up
C16orf54	transmembrane protein C16orf54	2.34	2.89	Up
FIS1	mitochondrial fission 1 protein	1.48	1.54	Up
RALY	RNA-binding protein Raly	1.5	1.91	Up
NPM1	nucleophosmin	1.59	1.64	Up
S100A4	protein S100-A4	0.67	3.51	Down / Up
HLA-E	HLA class I histocompatibility antigen, alpha chain E precursor	0.57	1.67	Down / Up
GMFG	glia maturation factor gamma	1.72	0.26	Up / Down
GMFB	glia maturation factor beta	1.82	0.55	Up / Down
PPP1R18	phostensin	1.71	0.39	Up / Down
SGPL1	sphingosine-1-phosphate lyase 1	1.8	0.53	Up / Down
ISYNA1	inositol-3-phosphate synthase 1	1.82	0.57	Up / Down

The differentially regulated proteins from the LC and ME/CFS study were compared to identify matches using protein name, GI number and protein symbol. While 1.5 was the fold change cut off PRDX2 and FIS1 were included as they are close to 1.5 fold.

For example, HLA-B, a protein that helps the immune system distinguish self-proteins from those of exogenous viruses and bacteria was significantly down-regulated in both LC group and the ME/CFS group. C16orf54, a transmembrane protein that is suggested to regulate homeostasis of cell energy supply was highly up-regulated in both LC and ME/CFS study groups. In addition to those that were regulated in the same direction for LC and ME/CFS, there were also proteins significantly regulated but in the opposite direction in the two datasets (not recorded in the Venn diagrams in Fig. 4 as overlapping because of their up/down directions). They are also shown in the table. For example, S100A4, a Ca^2+^binding protein expressed in CD4 + CD25 + lymphocytes is almost fourfold upregulated in the LC study group but 1.5 fold downregulated in ME/CFS study group. CD4^+^CD25^+^ immunoregulatory T cells represent a unique lineage of thymic-derived cells that potently suppress both in vitro and in vivo effector T cell function and allow tolerance to endogenous antigens to modulate an autoimmune response. Two glial maturation factors (GFM-B & CFM-G) are almost twofold up-regulated in the LC study group but 2–fourfold down-regulated in the ME/CFS group. GMFB, considered a growth and differentiation factor for both glia and neurons and associated with neuroinflammation is up-regulated, while GFMG is a cytokine responsive protein mainly expressed in inflammatory cells and regulates the chemotaxis of neutrophils and lymphocytes.

An important outcome of the original ME/CFS published study was that many mitochondrial proteins were identified. They were involved in both general functions, metabolism, electron transport complexes, and the reactive oxygen species stress response. The re-evaluated MCL cluster analysis of the ME/CFS differentially regulated proteins here identified a mitochondrial cluster of 13 proteins (Fig. 3E).

When the differentially regulated proteins from the LC dataset were searched against the Human MitoCarta3.0^44^ database of human mitochondrial associated proteins, 21 of the 162 proteins were identified as mitochondrial (Table 3). The LC differentially regulated proteins in the current study identified a small six protein cluster of mitochondrial proteins (Cluster 7, Supplementary Table S3).

**Table 3:** Differentially regulated mitochondrial proteins in the LC data.

Peak Name	Symbol	Protein name	FC	*p*-value	Activity (MitoPathways)
Mitochondrial Metabolism					
gi|373,251,164	GLS	Glutaminase	1.53	0.028	Glutamate metabolism
gi|937,827,788	LDHB	L-lactate dihydrog. B chain	1.57	0.011	Glyoxylate metabolism
gi|23,618,867	SFXN1	sideroflexin-1	1.70	0.002	Serine and Vitamin metabolism
gi|578,829,057	PDPR	pyruvate dehydrogenase (PDH) phosphatase regulatory subunit	2.85	0.013	Pyruvate metabolism
gi|767,969,704	DLAT	dihydrolipoyllysine-residue acetyltransferase (PDH complex)	1.64	0.042	Pyruvate metabolism
gi|32,189,392	PRDX2	peroxiredoxin-2	1.70	0.024	ROS and GSH metabolism
gi|8,923,001	ABHD10	mycophenolic acid acyl-glucuronide esterase	1.60	0.035	Xenobiotic metabolism
gi|395,394,071	TST	thiosulfate sulfurtransferase	0.64	0.010	Sulfur metabolism
gi|13,376,617	PTGES2	prostaglandin E synthase 2	1.75	0.027	Eicosanoid metabolism
gi|15,277,342	HSD17B8	estradiol 17-beta-dehydrogenase 8	2.43	0.018	Type II fatty acid Cholesterol, bile acid, steroid synthesis
gi|37,594,464	NUDT5	ADP-sugar pyrophosphatase	0.64	0.028	Nucleotide synthesis and processing
Mitochondrial Translation					
gi|38,683,855	PTCD3	pentatricopeptide repeat domain-containing protein 3, mitochondrial precursor	1.84	0.029	Mitochondrial ribosome;
gi|8,923,421	SARS2	seryl-tRNA synthetase 2, mitochondrial	2.07	0.008	mt-tRNA synthetases
gi|46,852,147	IARS2	isoleucyl-tRNA synthetase 2, mitochondrial precursor	1.51	0.040	mt-tRNA synthetases
gi|815,890,954	GFM1	elongation factor G, mitochondrial	0.45	0.044	Translation factors
Mitochondrial dynamics and surveillance					
gi|767,999,127	BCL2	apoptosis regulator Bcl-2	2.20	0.044	Apoptosis
gi|151,108,473	FIS1	mitochondrial fission 1 protein	1.54	0.025	Fission
Oxidative Phosphorylation—Complex I					
gi|7,661,786	NDUFAF4	NADH dehydrogenase [ubiquinone] 1 alpha subcomplex assembly factor 4	1.97	0.008	OXPHOS assembly factors
gi|4,758,784	NDUFC2	NADH dehydrogenase [ubiquinone] 1 subunit C2	0.48	0.015	OXPHOS subunits
gi|7,706,351	PTRH2	peptidyl-tRNA hydrolase 2, mitochondrial	0.46	0.001	none
gi|767,902,514	CRYZ	quinone oxidoreductase	1.56	0.010	none

The human MitoCarta3.0 database was accessed to search for proteins associated with the mitochondria within the LC data set.* FC* fold change.

From the 83 up-regulated proteins in the LC dataset (Fig. 1B) the 16 up-regulated mitochondrial proteins shown in Table 2 represent an enrichment that does not occur by chance. A binominal test using the binomial distribution range function (BINOM.DIST.RANGE) in Microsoft Excel resulted in a probability of *p* = 0.018 to identify 16 or more mitochondrial proteins from a pool of 83 up-regulated proteins. By contrast, the probability of finding the 5 down-regulated mitochondrial proteins shown in Table 3 amongst all 79 down-regulated proteins is 0.94, indicating that they could occur by chance. Pathway enrichment analysis of the 83 up-regulated proteins using the STRING database tools (https://string-db.org/) also showed an enrichment of proteins associated with the mitochondrion at a strength (log_10_(observed/expected)) of 0.41 and an adjusted *p*-value of 0.023 (Benjamini–Hochberg procedure) under the Gene Ontology (GO) aspect of Cellular Compartment, further supporting the conclusion that the mitochondrial proteins amongst the up-regulated proteins represents an enrichment.

## Discussion

The changes represented in the differential regulation of the proteins in the immune cells of PBMCs from the LC patients determined in this study were significant as indicated from the principal component analysis and were predominately focused in clusters whose functions were related to immune function, and gene expression. Not surprisingly, changes in proteins related to gene expression were highlighted as they would facilitate changes in expression that led to higher or lower amounts of proteins being synthesised. The results indicated that the immune system activity of LC patients one year after the onset of their COVID infectious illness was dramatically different from that of the healthy controls. It reflected a chronic dysfunctional state that had not been restored from the expected transient immune/inflammatory response mounted to cope with the original infection from the SARS-CoV-2.

The conclusions described here complement emerging studies with LC patients of changes in cellular immunology^22,45^. Immune profiling of 215 patients in conjunction with machine learning identified key features that differed from controls^45^. A study using multi-omics and serology compared LC patients with those who did not develop LC after their SARS-CoV-2 infection demonstrating systemic inflammation and immune dysregulation in the LC group. It was concluded the normal crosstalk between the humoral and cellular arms of adaptive immunity had broken down in the LC patients^46^. A further study up to 24 weeks post COVID revealed differences in innate immune cells natural killer cells, neutrophils, CXCR3 + monocytes, and in adaptive T cell populations^47^. LC patients have been shown to have reduced CD4 + and CD8 + effector memory cells^48^. Somewhat conflicting results have been found also in separate cohort studies of cytokines in LC^23,24,49^. Interleukin-1β, IL6 and TNα were elevated in those who did not recover from their viral infection indicating these three cytokines may have a significant role^23^.

Of the immune system proteins identified in the current study several human leukocyte antigen (HLA) proteins were identified as differentially regulated in both LC and ME/CFS datasets. HLA proteins are transmembrane proteins that bind and present peptide antigens for detection and appropriate immune response by T cells, macrophages and natural killer cells (NK)^50^ and their role is to aid distinguishing between self and non-self. HLA genes are highly polymorphic and some immunological diseases arise from genetic variation^51^. Class I and Class II HLA proteins are expressed on antigen presenting cells (B cells, macrophages, dendritic cells). Differential regulation of HLA-B (Class I) and HLA-E (Non-classical Class I) were common to LC and ME/CFS, but HLA-DRB1 (Class II) was differentially regulated in the LC data alone, and HLA-A (Class I), HLA-DQB1 and HLA-DPB1 (both class II) were also differentially regulated in the ME/CFS study. HLA-B was down-regulated in both LC (sixfold) and ME (1.7-fold) suggesting altered T cell recognition and reduced inhibitory signaling in Natural Killer (NK) cells in both disorders. By contrast HLA-E was down-regulated in ME (1.6-fold) but up-regulated in LC (1.7-fold). HLA-E specifically recognises CD94 expressed on Natural Killer (NK) cells^52^. HLA-E overexpression has been shown to negatively interfere with innate immune responses protecting cells from susceptibility to lysis by NK cell-mediated cytotoxicity^53^.

Cluster of Differentiation markers (with the prefix CD) are a plethora of antigens on the cell surface of leucocytes. For example, CD5 is a T-cell surface glycoprotein that negatively regulates T cell receptor signaling from the onset of T-cell activation. CD4, 5, 84 and 300LF were differentially regulated in the LC dataset. Upregulated in both LC and ME/CFS datasets, CD4 is involved in the early phase of T cell activation, CD5 regulates T cell/B cell interactions and inhibits T cell receptor mediated signaling from the onset of T cell activation. Downregulated in both datasets, CD84 enhances T cell and NK cell activation and cytokine activation, and CD300LF is an inhibitory receptor of myeloid cells.

Since LC is a specific example of a post-viral fatigue syndrome that develops from a specific triggering virus and has a symptom profile and clinical case definition very similar to ME/CFS, the collective term that has been given to post-viral fatigue syndromes from multiple triggers, we compared the LC dataset with an ME/CFS dataset we had derived earlier using the same mass spectrometry approach, re-analysing the dataset with the same statistical parameters used in the current study. Data from supplementary Table S4 in the ME/CFS proteomic study^29^ that listed all identified differentially regulated proteins was filtered to give the same stringency (*p*-value and fold change) as was used in this LC study. With these parameters there were ~ twofold more differentially regulated proteins (346) compared with the LC data set (162) and thereby a somewhat more complex pattern in their clustering patterns (compare Fig. 3 to Fig. 2). Cluster analysis from the ME/CFS data set (Fig. 3) identified two separate clusters related to the immune system and its processes, a large cluster related to gene expression/metabolism, and two smaller clusters related to mitochondrial energy production, and vesicle mediated transport as the LC data set.

Of the 34 Reactome categories enriched in the LC data, 22 were also enriched in our earlier ME/CFS data, whereas of the 32 enriched KEGG pathways in the LC data, five were also enriched in ME/CFS (supplementary Figure S1). This highlights pathways that are similarly affected while showing there are distinctions.

A feature of the original ME/CFS study had been the number of differentially regulated mitochondrial proteins. A search of the LC dataset for significantly differentially regulated mitochondrial proteins identified 21 that were involved in mitochondrial metabolism, translation, dynamics, and oxidative phosphorylation. Of the 54 differentially regulated mitochondrial proteins identified in the ME/CFS data set, 14 formed a cluster in the string analysis here (Fig. 3E), whereas there was no cluster with at least 10 proteins in the LC string analysis from the 21 mitochondrial proteins identified. Thirty-five of the differentially regulated mitochondrial proteins in the ME/CFS dataset were detected in the 3131-protein spectral library used to analyse the LC data set. Despite the fewer differentially regulated proteins in the LC dataset 17 of these had very similar fold changes in both LC and ME/CFS datasets (supplementary Table S7), despite only some meeting the statistical criteria for significance, with the others trending towards significance. This may reflect a wider variation among the LC patient in the levels of differential regulation among some of the mitochondrial proteins.

Peroxiredoxin-2 (PRDX2) is a mitochondrial antioxidant enzyme upregulated in both LC and ME/CFS datasets. PRDX2 reduces reactive oxygen species (ROS) by hydrolysing H_2_O_2_. Elevated levels of PRDX2 in our studies suggests there is increased ROS generation in both LC and ME/CFS immune cells. PRDX2 has been implicated in neurological disease due to aberrant management of ROS^54^. Peroxisomes are an independent organelle that metabolically interact with mitochondria but also play a role in ROS production and scavenging, and dysfunctional effects on mitochondria can affect peroxisomal physiology^55^. Peroxisomal dysfunction is linked with decreased levels of plasmalogens (a class of glycerophospholipids in cell membranes) that have been reported in ME/CFS^56^.

Another up regulated protein in both studies is PSMB9 (proteasome subunit beta type-9 proprotein) that has been linked to autoinflammation and immunodeficiency^57^. Expression of this gene is also increased in COVID-19 patients^58^.

### Limitations of the study

It should be noted the average duration time of the condition in the ME/CFS group was 16 years (Table 4), whereas the long COVID patients had a much shorter duration of their post-viral condition (1 year) so the two groups are at different stages of their ongoing conditions. Now, with so many LC cases synchronized in time as a result of the pandemic longitudinal studies will be possible to follow the course of the dysfunctional pathology of the conditions with time. This has not occurred with ME/CFS as there has been a continuous ‘drip feed’ of cases from endemic viruses like Epstein-Barr virus, and stressor triggers affecting individuals, as well as cases from boutique infectious disease outbreaks. It may be, despite the many similarities of the result from the two cohorts indicated in this study, that differences reflect the different timepoint since onset of the conditions in the respective cohorts.

**Table 4:** Cohort characteristics.

Clinical characteristics	Long COVID cohort	Long COVID control	ME/CFS cohort	ME/CFS control
Number	6	5	9	9
Median age	39	40	49	38
Sex	M = 1, F = 5	M = 1, F = 4	M = 4, F = 5	M = 3, F = 6
Median illness duration	1 year	N/A	16 years	N/A
Initial trigger	SARS-Cov-2	N/A	Infection (6), other (3)	N/A

These analyses are pilot studies with small cohorts of patients. Nevertheless, these patients are very well characterised and diagnosed by an ME/CFS expert clinician using the same clinical case definition. Our growing experience is that with appropriate statistics meaningful significant data can be obtained about the pathophysiology of the post stressor diseases for such small cohorts. Different molecular analyses have repeatedly shown consistent changes to biochemical pathways relating to the same dysfunctional physiology. Longitudinal studies with individual ME/CFS patients acting as their own controls have also been definitive. For example, in a relapse recovery cycle study molecular changes occurred during a relapse but then were restored to pre-relapse levels on recovery^32^.

*p*-value false discovery adjustment has not been included with the data presented here due to the low number of n (subjects) and a high biological variability between the subjects. A false discovery estimation using the Benjamini–Hochberg procedure resulted in only nine significant differences (adjusted *p* ≤ 0.05) between the study groups indicating a large number of false negatives after *p*-value adjustment. The scope of this study was to draw conclusions about biological effects and gain insights into potential mechanisms underlying LC in comparison to ME/CFS by performing a discovery proteomics approach. It was important to consider that false negative identifications from such comparisons would be lost for any data interpretation and may not be considered for any follow up studies, whereas potential false positives can be further tested for their significance by orthogonal methods.

## Conclusion

While this is a pilot study with only a small number of LC cases, we have found with multi-omic studies of well characterized ME/CFS patients that significant and meaningful results are forthcoming from such small cohorts when compared with age/sex matched controls, and the observed molecular changes are consistent across different molecular classes to detect common and expected pathophysiology^26,29,32^. Subgroups have been suggested for ME/CFS patients in multiple publications^59–62^. Indeed, we have examined individual patients by a precision medicine approach within a well characterized homogeneous small subgroup (age/sex, length of illness, relative functional activity levels) and have found individual patient differences in the molecular changes that still results in a very similar pathophysiology^6,21^. We believe this reflects the individual’s health history and genetic background that makes them susceptible to mount an inappropriate but immune response with individual variations to an external stressor that becomes chronic and spreads to involve the brain and CNS^14^, resulting in the ongoing neurological symptoms that characterise the condition. LC is known to be heterogeneous in that it contains those with ongoing organ damage from their SARS-CoV-2 infection as a well as a significant cohort of a classic post-viral fatigue syndrome like ME/CFS. Recently, four clinical phenotypes have been defined in a large LC cohort analysis, the major chronic fatigue-like condition (42%), a respiratory syndrome (~ 23%), a chronic pain syndrome (22%), and a neurosensorial syndrome (~ 11%)^5^.

This may help to explain conflicting results from the study of different cohorts of ME/CFS in the literature if there are different proportions with these phenotypes. It complicates understanding the holistic pattern of changes in the chronic immune system in LC (and in ME/CFS if there are indeed clinical phenotypes).

How does the occurrence of clinical phenotypes interfere with obtaining an accurate understanding of how closely related LC and ME/CFS are and on developing therapeutic strategies to improve the health of patients? Multiple papers have documented changes in both peripheral immune cell numbers and their activities, and the up or down regulation of cytokines^21^. While there may be individual differences dependent upon the viral trigger in LC or heterogeneous triggers in ME/CFS, this is likely also to reflect variations among the individual patients themselves according to their genetic background. The complexity is compounded as regulation can be in either direction according to the state and severity of the illness. Further coherence and integration to the myriad of changes is still required to provide more informed insight into what opportunities there are for therapeutic immune modulation in patients aimed at reversing the cascade of events that have led many to an ongoing severely debilitated state of health with LC, and for many years with ME/CFS. Potential therapeutic strategies to target immune mediated inflammatory diseases like LC and ME/CFS have progressed from broad specificity approaches to highly specific targeting of cytokines and their receptors and to small molecule drugs targeting the inflammatory pathways. Improving the quality of life for LC and ME/CFS patients generally might still be possible if the patient-to-patient variability in immune dysfunction is not too diverse.

## Methods

### Cohort recruitment

New Zealand LC patients who contracted the SARS-Cov-2 virus with the first wave of infection in March/April of 2020, and who had an ongoing fatigue illness with classical symptoms that were consistent with the clinical case definitions of ME/CFS^63,64^ were recruited with the help of a social media group of LC- affected and blood was collected between 8/3/21 and 12/4/21 together with age and sex matched healthy controls. The LC patients had contracted the virus ~ 12 months prior to sampling and been diagnosed with long COVID by an expert ME/CFS clinician (Dr. Rosamund Vallings) or their own doctor. The ME/CFS cohort from the previously published study^29^ were a well characterized group clinically who had been diagnosed also by Dr Vallings, and recruited pre-COVID pandemic for the study. Characteristics of the two groups are shown in Table 4.

Although the female to male ratios were different in the ME/CFS study compared with the LC cohort used in this study, we had found no PCA separation on the basis of gender or age in the ME/CFS cohort^29^, and the one male patient in the LC study here was within the PC3 cluster shown in Fig. 1A.

The study conformed to the ethics approval 17/STH/188 for patient studies from the Southern Health and Disability Ethics Committee of New Zealand. Patient information sheet was provided and informed consent was obtained from all participants. Consultation with Ngai Tahu Research Committee of the University of Otago was carried out before the beginning of this research to ensure our research gives effect to Te Tiriti o Waitangi. Research involving human research participants was performed in accordance with the Declaration of Helsinki.

### Blood collection and PMBC isolation

Blood (20 mL) was collected in BD Vacutainer™ blood collection tubes containing K2 EDTA 2. In a class 2 biological safety cabinet, blood was diluted twofold in PBS and 4 mL was added to tubes containing 3 ml Ficoll-Paque Plus. After centrifugation at 400×g in a swinging bucket centrifuge without braking, the plasma layer was removed, followed by the PBMC layer which was transferred to a tube, mixed gently with 3 volumes of PBS and centrifuged at 100×g for 10 min. The pellet was washed by gentle resuspension in 6 ml sterile PBS and centrifugated at 100×g for 10 min. Finally, the pellet was resuspended in PBS or 6 ml FBS containing 10% DMSO when stored in cryovials in liquid nitrogen until required. Then extensive washing of the cells in PBS to remove FBS proteins was carried out before subsequent protein analysis.

### Sample preparation

Proteins were extracted from PBMCs and digested with trypsin using the S-Trap-mini kit (ProtiFi, Fairport NY) according to the manufacturer’s instructions. In brief PBMCs were thawed in 50 μL of lysis buffer (100 mM triethylammonium bicarbonate (TEAB) pH 7.5, and 5% (w/v) sodium dodecyl sulphate (SDS) in water). Cells were lysed and homogenised by three consecutive vortex (10 s) and sonication (1 min in a sonication bath) steps. The homogenate was then supplemented with 100 U of benzonase. After incubation at 37 °C for 30 min, the cell lysates were centrifuged at 30,000×g for 30 min at 20 °C to remove insoluble material and cell debris. The supernatant containing the soluble protein fraction was recovered and an aliquot of each sample was used for the estimation of protein amounts using the BCA Protein Assay Kit (Thermo Scientific). A volume containing 100 µg of protein was taken from each sample, and subjected to reduction and alkylation of cysteines using 5 mM tris(2-carboxyethyl) phosphine (TCEP) and 10 mM iodoacetamide (IAM) before loading the samples on individual S-Trap mini units following the recommended protocol.

### Protein identification and quantification by SWATH-MS

Data-dependent acquisition (DDA) mass spectrometry of a pooled sample containing an equal amount of every sample was used to generate a comprehensive spectral library containing the peptide spectra of all identified proteins from all of the samples. To achieve greater protein identification, the pooled sample was subjected to peptide pre-fractionation using a high pH reversed-phase peptide fractionation kit according to the manufacturer’s instructions (Thermo Scientific). Each of the 11 fractions was then analysed in two technical replicates using DDA mass spectrometry on a 5600 + Triple Time-Of-Flight (TOF) mass spectrometer coupled to an Eksigent “ekspert nanoLC 415” uHPLC system (AB Sciex), as previously described^29^. For peptide/protein identification, the raw data files of all fractions and technical replicates were searched against the human reference sequence database (downloaded from https://www.ncbi.nlm.nih.gov/ on 29/03/2019), which contains 87,570 sequence entries, using the Protein Piolet software (version 4.5). Trypsin, carboxymethylcysteine and biological modifications were selected for a thorough search setup.

Each sample (from five healthy controls and six long COVID patients) was analysed in four technical replicates by data-independent acquisition (DIA) mass spectrometry using the SWATH-MS workflow according to the details described previously^29^.

### Data analysis and statistics

The spectral library was built using the SWATH application (version 2.0) embedded into PeakView software (version 2.2, AB Sciex), applying the criteria outlined in^29^. DIA spectral data were aligned to the library spectra at a false discovery threshold for peak picking of q = 0.01 in at least one sample. The peak area under the curve (AUC) was then exported to MarkerView software (version 1.2, AB Sciex) for further statistical analysis. The median value of the AUC of the four technical replicates was used to calculate the mean between the biological replicates of LC or HC samples for each peptide and protein. The mean peptide/protein values were then compared between disease and control groups using a 2-tailed Student’s t-test. The criteria for being considered significantly differentially regulated were ≥ 1.5-fold (Log2 fold change ≥ 0.58 or ≤  − 0.58) with a *p*-value of ≤ 0.05 (− log10(*p*-value) ≤ 1.3).

Regulated proteins were analysed by functional association network analysis using the STRING database tool version 12 (https://string-db.org/). MCL cluster analysis with a 1.5 inflation parameter returned clusters of functionally proteins and enrichment analysis of Gene Ontology (GO) terms enabled identification of any overrepresented biological processes or pathways within the data. The comparison of disease and control samples was performed to find further potentially regulated proteins that are functionally associated.

### Re-analysis of the ME/CFS date from Sweetman et al. 2020

A comparison of the LC data with a previously published ME/CFS data set generated using the same methods was attempted to ascertain if the two diseases shared any similarity. The list of significantly differentially regulated proteins from Sweetman et al.^29^ Supplementary Table 4 were filtered to retain only those with a fold change minimum of 1.5. These proteins were subjected to a STRING functional network analysis as described for the LC data set. Identification of any common differentially regulated proteins was achieved by comparing GI number, gene symbol and protein names from both datasets.

### Supplementary Information

Supplementary Information.

## Data Availability

The mass spectrometry proteomics data have been deposited to the ProteomeXchange Consortium via the PRIDE partner repository with the data set identifier PXD045508.
